# Shelph2, a bacterial-like phosphatase of the malaria parasite *Plasmodium falciparum*, is dispensable during asexual blood stage

**DOI:** 10.1371/journal.pone.0187073

**Published:** 2017-10-26

**Authors:** Alexandra Miliu, Maryse Lebrun, Catherine Braun-Breton, Mauld H. Lamarque

**Affiliations:** DIMNP, CNRS, Université de Montpellier, Montpellier, France; INSERM, FRANCE

## Abstract

During the erythrocytic cycle of the malaria parasite *Plasmodium falciparum*, egress and invasion are essential steps finely controlled by reversible phosphorylation. In contrast to the growing number of kinases identified as key regulators, phosphatases have been poorly studied, and calcineurin is the only one identified so far to play a role in invasion. *Pf*Shelph2, a bacterial-like phosphatase, is a promising candidate to participate in the invasion process, as it was reported to be expressed late during the asexual blood stage and to reside within an apical compartment, yet distinct from rhoptry bulb, micronemes, or dense granules. It was also proposed to play a role in the control of the red blood cell membrane deformability at the end of the invasion process. However, genetic studies are still lacking to support this hypothesis. Here, we take advantage of the CRISPR-Cas9 technology to tag *shelph2* genomic locus while retaining its endogenous regulatory regions. This new strain allows us to follow the endogenous *Pf*Shelph2 protein expression and location during asexual blood stages. We show that *Pf*Shelph2 apical location is also distinct from the rhoptry neck or exonemes. We further demonstrate *Pf*Shelph2 dispensability during the asexual blood stage by generating *Pf*Shelph2-KO parasites using CRISPR-Cas9 machinery. Analyses of the mutant during the course of the erythrocytic development indicate that there are no detectable phenotypic consequences of *Pfshelph2* genomic deletion. As this lack of phenotype might be due to functional redundancy, we also explore the likelihood of *Pf*Shelph1 (*Pf*Shelph2 paralog) being a compensatory phosphatase. We conclude that despite its cyclic expression profile, *Pf*Shelph2 is a dispensable phosphatase during the *Plasmodium falciparum* asexual blood stage, whose function is unlikely substituted by *Pf*Shelph1.

## Introduction

Apicomplexan parasites represent tremendous issues in terms of economy and public health concerns. *Plasmodium falciparum*, the etiologic agent of the deadliest form of malaria, is responsible for almost 500000 deaths every year, mostly affecting children in sub-Saharan Africa [1]. The lack of vaccine and the emergence of drug-resistant parasites highlight the urge to find new therapeutic targets.

*P*. *falciparum* life cycle alternates between its definitive host, the *Anopheles* mosquito, and the human host where it replicates asexually first in the liver, and then repetitively in the red blood cells (RBCs). The 48 hours (h) asexual blood cycle can be divided into several steps: RBC invasion, intracellular development within a vacuole, followed by several rounds of DNA replication that culminate in the individualization of new daughter cells named merozoites. Finally, these merozoites egress from the host cell and a new erythrocytic cycle can be initiated. Each one of these steps must be tightly regulated to ensure the release of mature merozoites fully competent for the next invasion step.

Egress and invasion take place within less than a minute and rely on a rapid burst of protein discharge from several apical organelles, including exonemes, micronemes and rhoptries. To orchestrate these finely tuned events, the parasite uses complex signaling pathways relying mainly on cyclic nucleotides, namely cyclic guanosine monophosphate (cGMP) and cyclic adenosine monophosphate (cAMP), and calcium as signaling molecules [2–5]. These signals activate their dedicated kinases, i.e. *Pf*PKG, *Pf*PKA and calcium-dependent protein kinases (CDPKs), that will in turn phosphorylate their respective substrates thought to be direct or indirect effectors required for these steps to proceed [3,5–11].

In sharp contrast to kinases, phosphatases (PPs) have received much less attention so far. Yet, a recent functional analysis of the murine malaria model *P*. *berghei* phosphatome revealed that out of 30 PPs, 16 of them are likely essential during asexual stages while another 6 are required for full sexual development in the mosquito, thus highlighting the crucial importance of these enzymes for parasite survival [12]. No such analysis has been conducted yet in *P*. *falciparum*, and calcineurin is the only PP reported to be involved in invasion based on reverse genetic studies [13].

In a search for *P*. *falciparum* PPs potentially involved in egress/invasion, we focused our interest on two bacterial-like PPs on the basis of their transcriptomic profile [14]. They are more closely related to PPs from bacteria of the *Schewanella* genus and therefore named *Pf*Shelph1 (PF3D7_1469200) and *Pf*Shelph2 (PF3D7_1206000) for “*Schewanella*-like phosphatase” [15,16]. Apart from bacteria, Shelphs are only found in Chromoalveolata, Fungi, Plantae and parasites belonging to Excavata, making them attractive putative targets for drug development [15,17]. Both *shelph* genes have been independently knocked-out in *P*. *berghei*: the loss of *Pb*Shelph2 did not disturb the parasite life cycle neither in mice nor in mosquitoes, while the absence of *Pb*Shelph1 affected ookinete development and consequently parasite transmission [12,17]. In *P*. *falciparum*, Fernandez-Pol *et al*. reported a perinuclear localization of *Pf*Shelph2 in schizonts and merozoites, distinct from microneme, rhoptry and dense granule markers [18]. They also determined that *Pf*Shelph2 displays a restricted tyrosine phosphatase activity *in vitro*, as was reported for *Pb*Shelph1 [17,18]. Therefore, despite being grouped within the serine/threonine PhosphoProtein Phosphatase (PPP) group, based on the presence of consensus signature motifs in their protein sequences [16,17], *Plasmodium* Shelphs exhibit the same specificity as their bacterial ortholog which is a strict tyrosine phosphatase [19]. Although reverse genetic studies are still lacking for *P*. *falciparum* Shelphs, two functional association studies, based primarily on co-transcriptional profile analyses, identified *Pf*Shelph2 as a protein that might be involved in invasion because of its co-expression with known “invasome” genes [20,21]. Furthermore, the enzyme was shown to dephosphorylate Band3 *in vitro*, one of the major surface protein of red blood cells [18]. As Band3 tyrosine phosphorylation status is known to control its association/disassociation from the RBC sub-membranous cytoskeleton [22], it has been hypothesized that *Pf*Shelph2 might modulate band3 phosphorylation during the course of invasion. Yet, so far no direct evidence of *Pf*Shelph2 function during the asexual blood stage has been established.

In this study, we undertook the functional characterization of *Pf*Shelph2 during the RBC cycle using reverse genetics. We tagged the endogenous gene using CRISPR-Cas9 technology to characterize *Pf*Shelph2 expression at the protein level and showed that it was strictly restrained to late schizonts and merozoites. Its location at the merozoite apex did not colocalize with rhoptry neck or exoneme markers. We also successfully generated a Shelph2-KO line, thereby demonstrating that this PP is dispensable during asexual development. The phenotypic characterization of the mutant revealed that the absence of Shelph2 did not induce any significant defect during the erythrocytic cycle, suggesting possible functional redundancy. Finally, we explored whether *Pf*Shelph1 might be a compensatory PP for *Pf*Shelph2 loss and conclude that this hypothesis is very unlikely.

## Materials and methods

### Parasite culture and transfection

*P*. *falciparum* 3D7 strain, obtained from the Malaria Research and Reference Reagent Resource Center (MR4-BEI resources, MRA-102), was cultured in human erythrocytes obtained as donations from anonymized individuals from the french Bloodbank (Etablissement Français du Sang, Pyrénées Méditerranée, France) at 5% hematocrit in RPMI 1640 medium (Gibco), supplemented with gentamycin at 20 μg/ml and 10% human serum [23]. The cultures were kept at 37°C under a controlled trigaz atmosphere (5% CO_2_, 5% O_2_ and 90% NO_2_). For synchronization, mature parasites were isolated using gelatin floatation [24]. Alternatively, late schizonts were collected on cushions of 70% (v/v) Percoll adjusted to isotonicity [25]. To restrict the invasion time-frame, parasites were subsequently synchronized in ring stages using 5% sorbitol [26].

For *Pf*3D7 transfections, 5–10% ring stages were transfected with 60–80 μg of circular or linear plasmid DNA as described previously [27,28]. Transgenic parasites were grown in agitation (200 rpm) and selected by addition of 2.5 nM WR99210 (for pL7-Shelph2*-HA_3_, pL7-Shelph2-KO or pARL2-Shelph1-GFP), and 1.5 μM DSM1 (for pUF1-Cas9). Drug pressure was removed after parasite genotyping, except for the maintenance of pARL2-Shelph1-GFP.

### Molecular biology

All the primers used in this study are listed in S1 Table. All the PCR products have been verified by sequencing (Eurofins).

The PCR reactions were performed using the Phusion or Q5 DNA polymerase (NEB Biolabs). Bacterial colonies were screened using the GoTaq G2 Green master mix (Promega).

RNA extraction was performed using the NucleoSpin® RNAII kit (Macherey-Nagel). cDNA preparations were obtained by reverse transcription using Superscript III First-Strand Synthesis SuperMix for RT-PCR (Invitrogen) and 500 ng—1 μg of total RNA.

For qRT-PCR, two independent RNA samples from *Pf*3D7 and the three *Pf*Shelph2-KO lines were prepared. Each cDNA was diluted 1/20 before measuring by qPCR. *shelph1* (PF3D7_1469200), *PPKL* (protein phosphatase containing kelch-like domains; PF3D7_1466100), and *shelph2* mRNA expression were quantified using the LightCycler 480 Sybr Green I system (Roche) using primers listed in S1 Table. Fructose-biphosphate aldolase (FBA; PF3D7_1444800) was used as the reference gene. LightCycler 480 Software version 1.5 was used for relative quantification analysis. The expression of each target gene in *Pf*Shelph2-KO was then normalized to *Pf*3D7 expression level.

### Plasmid constructs

To generate pL7-Shelph2*-HA_3_ vector, we first amplified the triple HA tag from pLIC-DHFR [29] using primers MLa33/MLa32 and cloned it SpeI/AscI into pL6_BsgI_V3 (modified version of pL6_eGFP, gift from Jose-Juan Lopez-Rubio). This generated pL6_BsgI-HA_3_ plasmid. Next, we amplified 646 bp of *shelph2* 3’UTR from *Pf*3D7 gDNA using primers MLa40/MLa41 and cloned it AscI/SacII into pL6_BsgI-HA_3_ to generate pL6_BsgI-HA_3_-3’UTR. The 3’UTR was designed 207 bp downstream of *shelph2* stop codon due to a very rich A/T richness that prevented the design of a specific primer. The full *shelph2* coding sequence (CDS) amplified using primers MLa3/MLa4 was first subcloned into the pCR-BluntII-TOPO vector (Invitrogen). Shield mutations in *shelph2* CDS were introduced by mutagenesis with primers MLa79/MLa80 using the QuickChange Site-directed Mutagenesis kit (Stratagene) according to the manufacturer instructions. The resulting mutated shelph2* was again subcloned into the pCR-BluntII-TOPO and verified by sequencing. 712 bp of shelph2* was re-amplified using primers MLa59 and MLa45 and cloned into pL6_BsgI-HA_3_-3’UTR using SpeI, yielding pL6_BsgI-shelph2*-HA_3_-3’UTR. From this vector, the whole shelph2*-HA_3_-3’UTR cassette was re-amplified using primers MLa59/MLa60 and cloned InFusion (Clontech) SpeI/AflII into pL6-eGFP [28]. The resulting vector pL6-shelph2*-HA_3_-3’UTR was digested BtgZI to allow the insertion of *shelph2* gRNA corresponding to hybridized primers MLa63/MLa64. The final plasmid named pL7-Shelph2*-HA_3_ was used for transfection.

To generate pL7-Shelph2-KO vector, 388 bp fragment encompassing the 5’UTR and the first 219 bp of *shelph2* CDS was amplified by PCR as homology region 1 using primers MLa54/MLa53. The fragment was cloned NcoI/EcoRI by InFusion into the pL6-eGFP vector, downstream of hDHFR cassette, giving pL6-3’UTR. Similarly, a 760 bp fragment corresponding to *Pfshelph2* 3’UTR was amplified using primers MLa50/MLa51 and cloned AflII/SpeI by InFusion into pL6-3’UTR plasmid, upstream of hDHFR cassette. Finally, gRNA MLa63/MLa64 was inserted into the plasmid in BtgZI as described above. The resulting vector was named pL7-Shelph2-KO and used to transfect parasites.

To generate pARL2-Shelph1-GFP plasmid, the entire *shelph1* coding sequence without the stop codon was PCR amplified using primers MLa1 and MLa2 and cloned XhoI/KpnI in frame with a GFP tag into pARL2-GFP vector [30].

### *Pf*Shelph2-KO phenotypic assays in asexual blood stage

All the experiments described below have been performed on tightly synchronized parasites with a 2 hours re-invasion time frame.

To follow *P*. *falciparum* intra-erythrocytic development, synchronized parasite cultures were smeared in triplicate from 2h post-invasion until 48h. The ratio of ring, trophozoite and schizont was evaluated for 200 infected RBCs at each time point.

For determining the number of merozoites per segmenter, late schizonts of about 40h were purified on a Percoll gradient, and parasites were left maturing for an additional 4h in the presence of 1.5 μM compound 2 to block egress [6,31]. After one wash in complete medium, blood smears were done in triplicate and analyzed by counting 50 segmenters per smear.

Proliferation rate assays were set up at 1% parasitemia in ring stage. Samples were taken up in ring stage 6h post-invasion during the first cycle. 48h later, ring stage samples of the next cycle were again collected and fixed in 4% paraformaldehyde (PFA) for 4h at room temperature (RT). Flow cytometry (FACS) was then used to determine the parasitemia. Fixed cells were washed twice in phosphate buffer saline (PBS), followed by 30 minutes (min) incubation with 1X SybrGreen (Invitrogen) in the dark. Cells were washed, resuspended in 700 μl PBS and analyzed by BD FACS Canto I flow cytometer using FACS Diva software (BD Biosciences). SYBR green was excited with a blue laser at 488 nm, and fluorescence was detected by a 530/30 nm filter.

### Immunoblot and immunofluorescence assays

Immuno-fluorescence assays (IFAs) were performed on smeared infected RBCs. Cells were fixed with 4% PFA for 30 min at RT before a 10 min permeabilization step in PBS-0,1% Triton X100. Following 30 min saturation in PBS-1.5% bovine serum albumin (BSA), cells were incubated 45 min with primary antibodies diluted in PBS-BSA. After 3 washes in PBS, cells were incubated 45 min with secondary Alexa-488- or Alexa-594-conjugated secondary antibodies highly cross-adsorbed (Invitrogen) diluted in PBS-BSA as recommended by the manufacturer. Nuclei were stained with Hoechst. Images were taken on a Zeiss Axioimager Z2 equipped with an apotome, at the Montpellier RIO imaging facility. Images were processed by Zen Blue edition software (Zeiss) for optical sectioning, luminosity and contrast adjustment. Antibody dilutions were rat anti-HA 1/100 (Roche Diagnostics), mouse anti-MSP1 19kDa 1/1000 (gift from M. Blackman), mouse anti-PfRON4 1/100 [32] and mouse anti-SUB1 pure (gift from M. Blackman).

Proteins were analyzed by SDS-PAGE and immunoblot. When required, parasites were separated from RBC by 0.01% saponin treatment for 5 min at 4°C prior to resuspension in Laemmli sample buffer in reduced conditions. Antibody dilutions were rat anti-HA 1/100 (Roche Diagnostics), rabbit anti-Histone H3 1/10000 (Abcam). Secondary antibodies conjugated to alkaline phosphatase (Promega) were diluted according to the manufacturer’s instructions and used with NBT/BCIP reagents (Promega).

## Results and discussion

### *Pf*Shelph2 biosynthesis starts during late schizogony, and its apical positioning is distinct from rhoptries or exonemes

**Fig 1:**
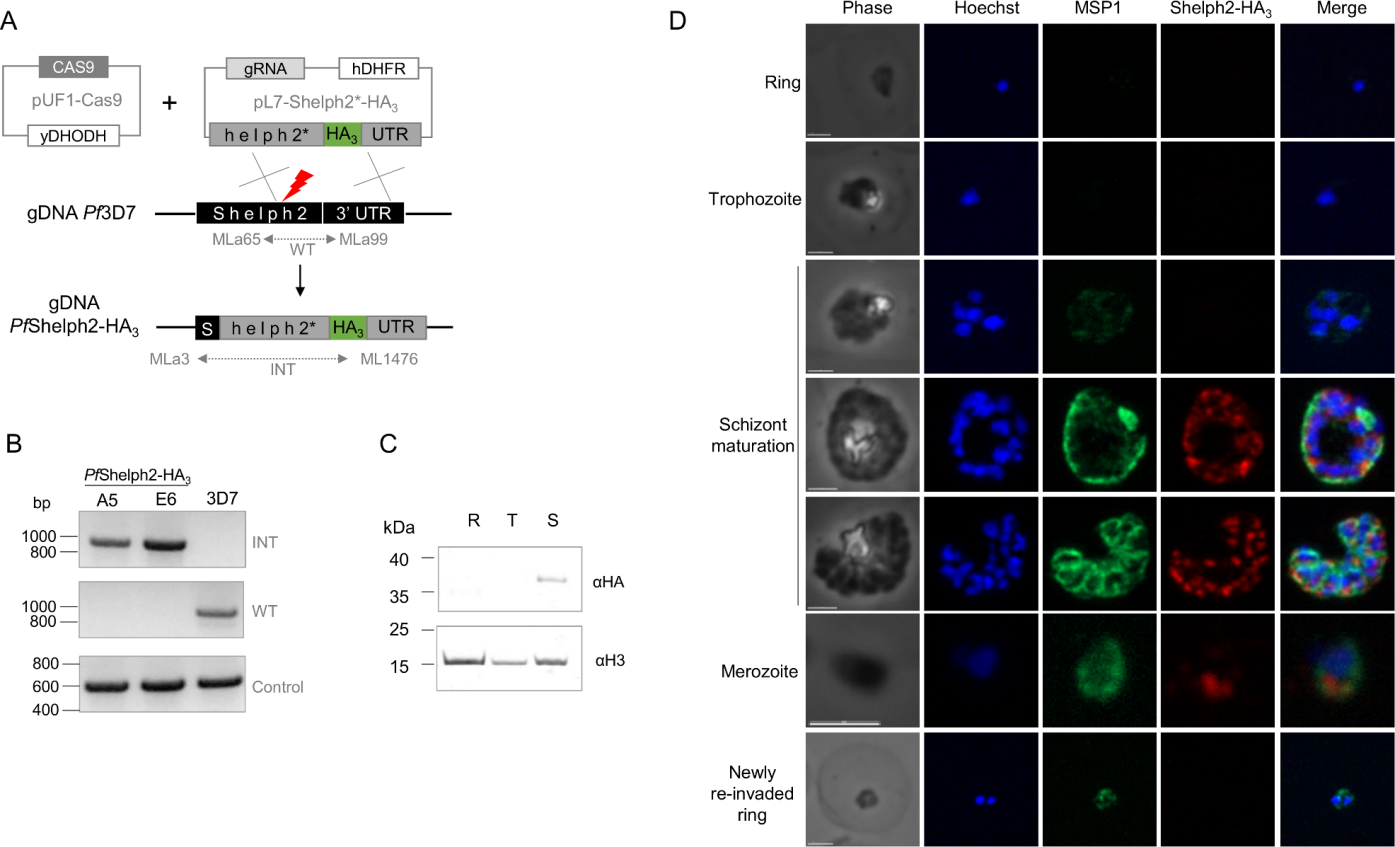
Transgenic *Pf*Shelph2-HA_3_ characterization. (A) Scheme depicting the strategy used to engineer *Pf*Shelph2-HA_3_ parasites. Plasmids pUF1-Cas9 and pL7-Shelph2*-HA_3_ were transfected in *Pf*3D7 and selected by addition of DSM1 and WR drugs respectively. The red thunder represents Cas9 double strand break. Parasites were genotyped by PCR (WT and INT) using primers depicted as arrows in the figure. (B) PCR genotyping of two edited *Pf*Shelph2-HA_3_ lines versus *Pf*3D7 parental strain. Control PCRs were performed on *Pf*PRL gene (PF3D7_1113100) using primers MLa11 and MLa12. (C) Immunoblot of *Pf*Shelph2-HA_3_ parasites on rings (R), trophozoites (T) or schizonts (S) extracts using anti-HA antibodies. Equivalent amounts of proteins were loaded per lane and verified using anti-Histone H3 antibodies. (D) IFA of *Pf*Shelph2-HA_3_ parasites using anti-HA and anti-MSP1_19_ antibodies at different stages of the RBC cycle, i.e. ring, trophozoite, schizont and merozoite. Scale bar, 2 μm.

We were interested in identifying PPs potentially involved in *P*. *falciparum* egress or invasion. In this context, *Pf*Shelph2 was selected as a candidate based on its transcriptional profile [14] and its putative role as an invasion protein [18,20,21]. To determine its protein expression profile in its native genomic context, we engineered a triple hemagglutinin (HA_3_) epitope tagged line using CRISPR-Cas9 technology [28] (Fig 1A). Following *Pf*3D7 transfection, parasites were selected using WR and DSM1, before cloning by limiting dilution. Two *Pf*Shelph2-HA_3_ clonal lines were analyzed by PCR for *shelph2* editing (Fig 1B). Integrative PCR (INT) confirmed the tag insertion in the two edited clones (A5 and E6), while the presence of wild type *shelph2* locus was detected with PCR WT in the parental line but not in *Pf*Shelph2-HA_3_ parasites. Sequencing of the *shelph2* locus confirmed the presence of the shield mutations in *shelph2* CDS and the in frame fusion of the HA_3_ tag at the C-terminus (S1 Fig).

We used *Pf*Shelph2-HA_3_ parasites to investigate *Pf*Shelph2 expression during the 48h erythrocytic cycle. Parasites were synchronized and samples were collected at ring, trophozoite and schizont stage for immunoblot (Fig 1C). Our results show that *Pf*Shelph2-HA_3_ migrated as a 37 kDa fusion protein, consistent with its predicted molecular mass after cleavage of its putative N-terminal signal peptide. Concordant with the transcriptomic data and previous immunofluorescence studies [14,18], the protein is only expressed in schizonts. Interestingly, a very recent ChIP-seq analysis identified *Pfshelph2* promoter region as a putative target of *Pf*AP2-I transcription factor [33]. *Pf*AP2-I is only expressed in trophozoites and schizonts and binds gene promoters that are predominantly invasion-related, chromatin-related and cell-cycle-related. This suggests that *Pf*Shelph2 late expression profile might rely on *Pf*AP2-I activity.

We also investigated *Pf*Shelph2 stage-specific expression by IFA, using Merozoite Surface Protein 1 (MSP1) staining as a marker of parasite maturation. MSP1 is one of the major merozoite surface components and is cleaved at a juxtamembrane position by a protease named *Pf*SUB2 [34], shedding the bulk of the protein while a 19 kDa fragment (MSP1_19_) is carried into the newly invaded parasite before being degraded [35]. As shown in Fig 1D, *Pf*Shelph2-HA_3_ is not detected from ring stage to young schizont. Then, during late schizogony, when MSP1_19_ gives either a peripheral schizont staining or labels the periphery of each merozoite during budding, *Pf*Shelph2-HA_3_ is found in discrete apical foci and remains detectable in free merozoites. In contrast, in newly re-invaded ring, evidenced by the persistent parasite membrane staining with MSP1_19_, *Pf*Shelph2-HA_3_ staining disappears. Our observations are consistent with the reported localization of *Pf*Shelph2 [18,20] and confirm the late biosynthesis profile seen by western-blot. The fact that the protein is detected in the same structure in late schizonts and in free merozoites, but not in newly invaded parasites, suggests that the protein might be secreted post-merozoite release, i.e. likely during invasion. This observation is in agreement with previous findings regarding *Pf*Shelph2 dynamics of release during the course of invasion, which showed using live video microscopy that its apical pattern was retained during the invasion step until the parasite was approximately halfway through the invasion process [18].

**Fig 2:**
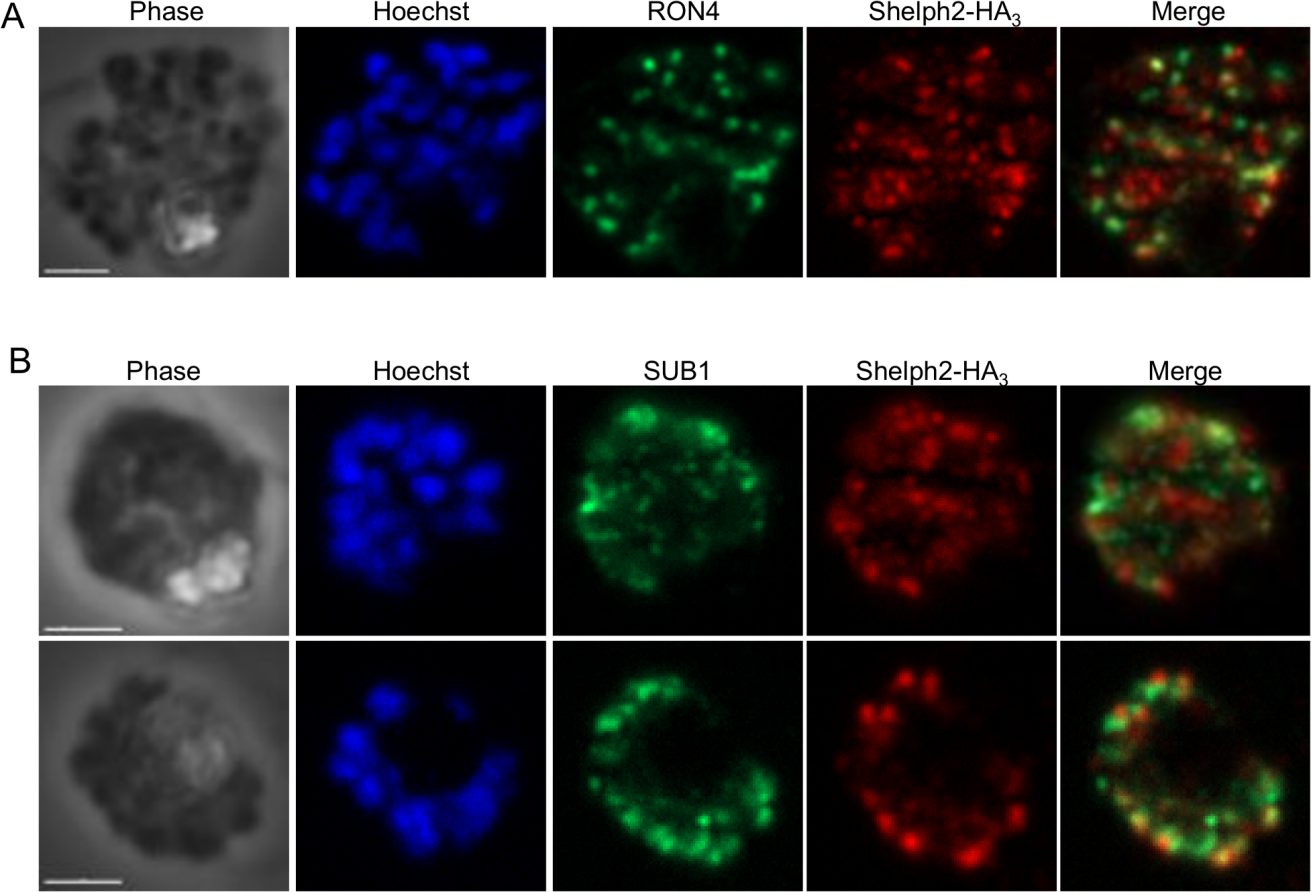
Dual labeling of *Pf*Shelph2-HA_3_ with rhoptry neck and exoneme markers. IFA of *Pf*Shelph2-HA_3_ parasites using anti-HA and (A) anti-*Pf*RON4 or (B) anti-*Pf*SUB1 antibodies on schizonts. Scale bar, 2 μm.

Given its apical localization in merozoites and its possible secretion during invasion, dual labeling with microneme, rhoptry or dense granule markers has been previously tested but failed to assign *Pf*Shelph2 to any of these organelles [18]. In the same study, *Pf*RON3, the ortholog of *Toxoplasma gondii* rhoptry neck protein 3, was used as a rhoptry neck marker. Yet, PfRON3 was shown to be a rhoptry bulb protein in *P*. *falciparum* [36,37]. This prompted us to re-analyze the possible association of *Pf*Shelph2 with this rhoptry sub-compartment using anti-*Pf*RON4 antibodies [32], but we found no obvious overlap between *Pf*Shelph2 and *Pf*RON4 signals in schizonts (Fig 2A). We then investigated whether *Pf*Shelph2 could be stored in exonemes. Exonemes resemble dense granules but are slightly more elongated organelles [38]. They have been identified by immuno-electron microscopy while investigating the subcellular localization of the serine protease *Pf*SUB1. *Pf*SUB1 is discharged in the parasitophorous vacuole just prior egress, and therefore, exonemes are considered as secretory organelles. We observed a very distinct pattern between *Pf*SUB1 and *Pf*Shelph2-HA_3_, leading us to conclude that *Pf*Shelph2 is not contained within exonemes (Fig 2B). Therefore, *Pf*Shelph2 displays an expression profile similar to many invasion-related proteins, and is located in an apical compartment in the invasive merozoite that is different from the rhotry neck or exonemes. Unfortunately, *Pf*Shelph2 expression level by IFA was too low to explore its localization further by immuno-electron microscopy.

### *Pf*Shelph2 is a dispensable PP during asexual blood stage

**Fig 3:**
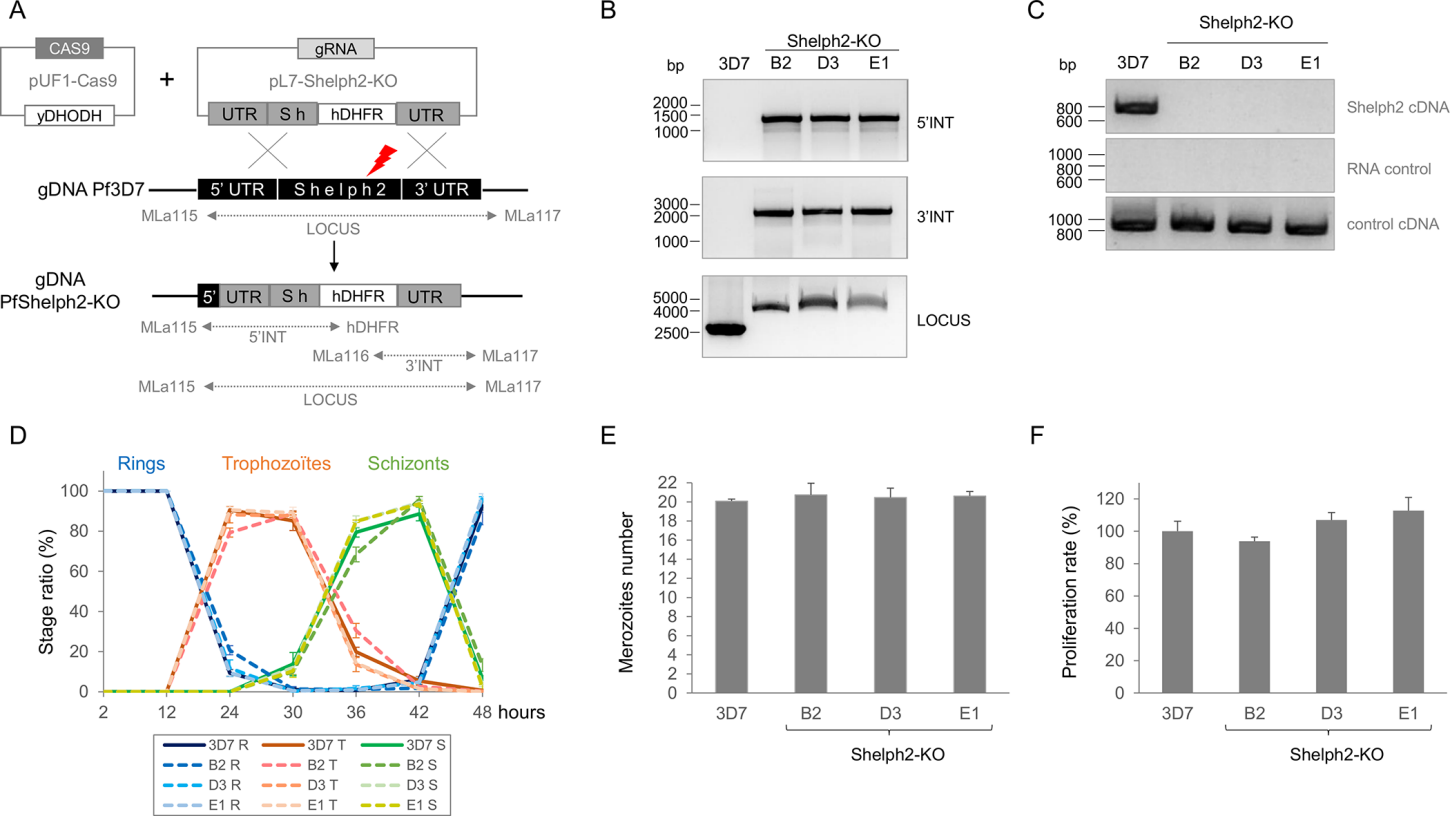
Successful disruption of *Pfshelph2* gene and phenotypic characterization of *Pf*Shelph2-KO parasites in asexual blood stage. (A) Scheme depicting the strategy used to generate *Pf*Shelph2-KO parasites. Plasmids pUF1-Cas9 and pL7-Shelph2-KO were transfected in *Pf*3D7 and selected by addition of DSM1 and WR drugs respectively. The red thunder represents Cas9 double strand break. Three clonal lines, namely B2, D3 and E1, were genotyped by PCR (LOCUS, 5’INT and 3’INT) using primers depicted as arrows in the figure. (B) PCR genotyping of three *Pf*Shelph2-KO lines versus *Pf*3D7. PCR 5’INT refers to 5’ integration, PCR 3’INT to the 3’integration, while PCR LOCUS shows the amplification of the whole *shelph2* genomic locus. (C) RT-PCR of *shelph2* mRNA in *Pf*Shelph2-KO lines versus *Pf*3D7. RNA samples were also used as PCR templates to verify the absence of contaminating genomic DNA. Control cDNA corresponds to the 880 bp amplification of PF3D7_1127000 from cDNA using primers MLa13 and MLa14, while PCR from gDNA is expected to amplify a 1850 bp fragment. (D) The intra-erythrocytic development of *Pf*Shelph2-KO lines B2, D3 and E1 was followed in comparison with *Pf*3D7. For each time point, smears were done in triplicate and 200 infected RBCs were counted per smear. The graph shows the ratio of each stage (R for ring, T for trophozoite and S for schizont) as a function of time post-invasion. Error bars represent standard deviation. This experiment was repeated 2 times independently. (E) The graph represents the number of merozoites produced per schizont in *Pf*3D7 or *Pf*Shelph2-KO lines. Tightly synchronized schizonts of about 40h were allowed to mature in presence of compound 2 for 4h. After one wash in complete medium, smears were done in triplicate and 50 infected RBCs were counted per smear. Error bars represent standard deviation. This experiment was repeated 3 times independently. (F) Parasite proliferation rate was measured in ring stage by FACS from one cycle to the next one. Results are shown as a percentage of *Pf*3D7 proliferation rate. Error bars represent standard deviation. This experiment was repeated 3 times independently.

Next, we investigated *Pf*Shelph2 function during the red blood cell cycle by a direct knock-out strategy where we aimed to replace most of *shelph2* CDS with the selectable hDHFR marker (Fig 3A). We isolated three *Pf*Shelph2-KO clonal lines (B2, D3 and E1) that were genotyped by PCR for hDHFR cassette integration (Fig 3B). We also confirmed the absence of *shelph2* mRNA by RT-PCR (Fig 3C).

Having established *Pf*Shelph2-KO lines, we next assessed the possible phenotypic consequences of *Pf*Shelph2 loss during the asexual blood stage. First, we followed their intracellular development. To do this, parasites were tightly synchronized within a 2 hours window and ring-stage parasites were monitored through the cell cycle (Fig 3D). Progression from ring to trophozoite, evidenced by the detection of the hemozoin pigment, occurred around 24h, then followed by DNA replication during schizogony between 30-36h. Merozoites egress from host cell started from 42h and allowed the next cycle to take place as evidenced by the concomitant increase in rings. Thus, maturation of *Pf*Shelph2-KO parasites within the erythrocyte is not significantly altered compared to *Pf*3D7.

To assess for specific defects during schizogony, we next evaluated the parasite replication by counting the number of merozoites produced per schizont (Fig 3E). Again, synchronized cultures were allowed to progress until 40–42 h, at which time, parasites egress was blocked for 4h by *Pf*PKG inhibition in presence of compound 2 while not affecting schizonts maturation to segmenters [6]. All the strains produced an average of 20 merozoites per schizont, demonstrating that the absence of *Pf*Shelph2 does not affect mitosis or daughter cells formation. We then tested the mutant for possible subtle defects in egress/invasion. For this, newly re-invaded ring stages were allowed to progress to the next erythrocytic cycle and parasitemia was measured by FACS (Fig 3F). The results show that *Pf*Shelph2-KO growth rate is very similar to that of *Pf*3D7, suggesting that *Pf*Shelph2 does not play a prominent role neither in egress nor in invasion.

Despite its tightly controlled expression, our results demonstrate that *shelph2* knock-out does not affect *P*. *falciparum* intra-erythrocytic cycle as parasites maturation, daughter cell formation and proliferation rate were comparable to those of *Pf*3D7. These results are consistent with data obtained in *P*. *berghei*, where *Pb*Shelph2-KO did not impair neither asexual blood stage in mice, nor sexual development in mosquitoes [12]. Therefore, Shelph2 is a dispensable PP in *Plasmodium* species that can be explained either by a non-critical function, or by compensation by another PP. Functional redundancy during asexual blood stage was suggested in *P*. *berghei*, as 14 independent PP knock-outs, including *Pb*Shelph2-KO, did not induce any growth phenotype of the mutants in mice [12]. The most obvious compensatory PP for *Pf*Shelph2-KO line would be *Pf*Shelph1 that shares 29.5% similarity at the protein level and the same *in vitro* phosphatase specificity [17]. Although its function has never been investigated in *P*. *falciparum*, its ortholog *Pb*Shelph1 is critical for ookinete development and differentiation, thus demonstrating that at least in the mosquito, *Pb*Shelph2 cannot compensate for *Pb*Shelph1 loss [17].

**Fig 4:**
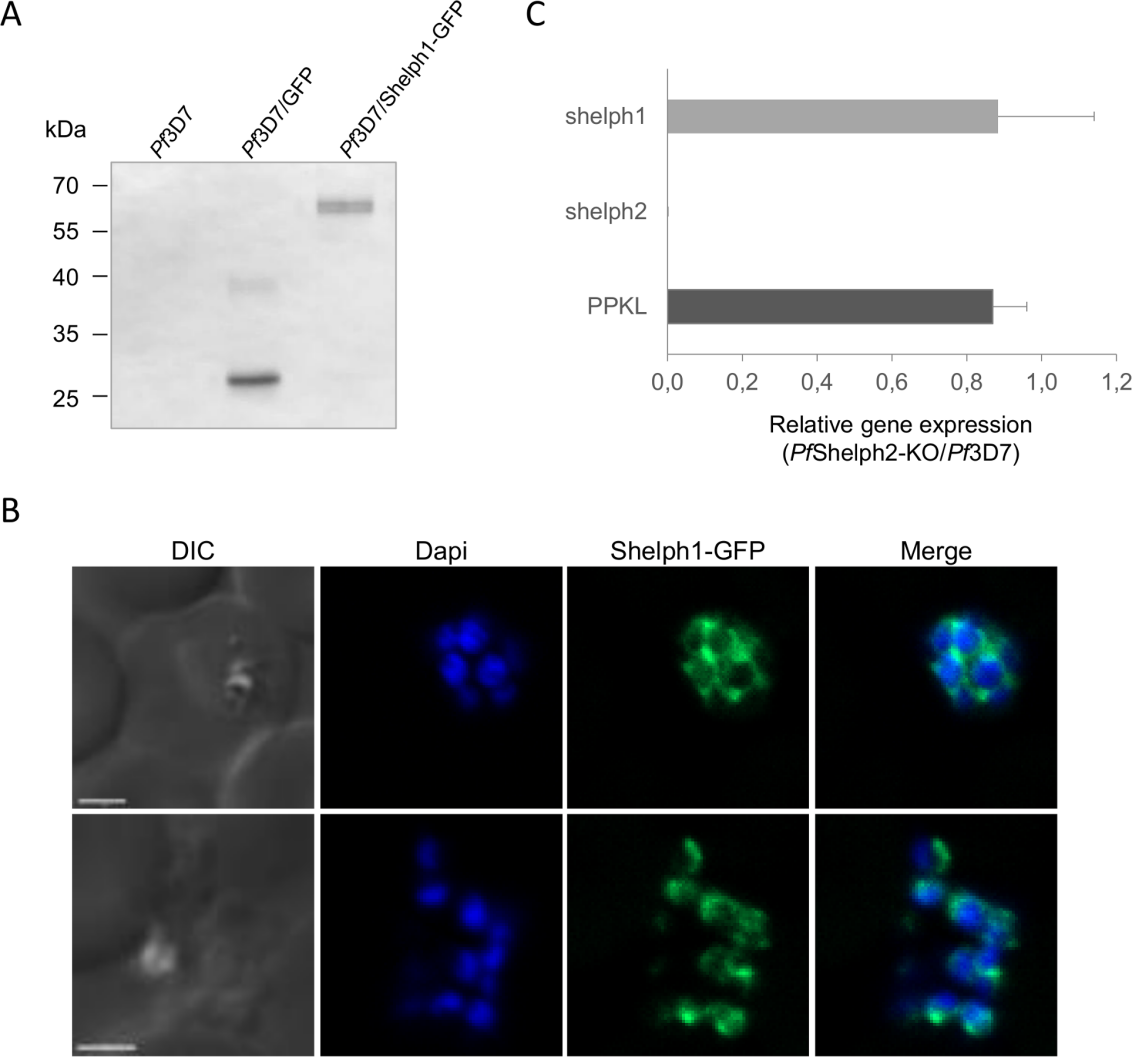
*Pf*Shelph1 localization and gene transcript levels. (A) Western-blot of *Pf*3D7, *Pf*3D7/GFP (empty vector) and *Pf*3D7/Shelph1-GFP parasite extracts. GFP alone is observed at 27 kDa, while Shelph1-GFP is expressed as a 66 kDa fusion protein. (B) Fluorescence microscopy of *Pf*3D7/Shelph1-GFP line. Scale bar, 2 μm. (C) Quantitative RT-PCR analysis was performed on cDNA from *Pf*3D7 and *Pf*Shelph2-KO parasites. The gene relative expression in *Pf*Shelph2-KO parasites is given following normalization to *Pf*3D7. *PfPPKL* and *Pfshelph2* transcript levels were used as controls. Fructose bisphosphate aldolase was used as the reference gene. The graph represents the mean of two independent experiments, for which the data of the three *Pf*Shelph2-KO lines were pooled. Error bars represent standard deviation.

To evaluate whether *Pf*Shelph1 might counterbalance the absence of *Pf*Shelph2, we first assessed its location by expressing a *Pf*Shelph1-GFP fusion ectopically in *Pf*3D7 parasites. By western-blot, the fusion protein is detected around 65 kDa, which corresponds to its expected molecular mass (Fig 4A). By fluorescence microscopy, we detected a perinuclear labeling during schizogony, suggestive of an endoplasmic reticulum positioning (Fig 4B). Although further co-localization studies should be done to properly refine this cellular location, our results would be concordant with the reported localization of *Pb*Shelph1 [17]. While the different locations of the two *P*. *falciparum* Shelphs do not point towards functional compensatory mechanism between the two enzymes, we ascertained this hypothesis by comparing *Pfshelph1* mRNA expression in *Pf*Shelph2-KO versus *Pf*3D7 by qRT-PCR. We used *Pfshelph2* mRNA as a negative control for the mutant, and *PfPPKL* phosphatase mRNA as a putative steady control based on the gene dispensability during asexual proliferation in *P*. *berghei* [39]. As anticipated, *Pfshelph2* mRNA becomes undetectable in *Pf*Shelph2-KO while *PfPPKL* expression is not significantly changed (Fig 4C). Likewise, we do not observe any major transcriptional regulation of *Pfshelph1*. Altogether, our results do not argue in favor of *Pf*Shelph1 being a compensatory PP in *Pf*Shelph2-KO, although further experiments should be done to properly assess this possibility.

*Pf*Shelph2 enzymatic activity as a tyrosine phosphatase is intriguing, as no canonical tyrosine kinase family has been described in *P*. *falciparum* [40]. Nevertheless, trace amounts of phospho-tyrosine phosphorylations (about 0.5%) have been detected in global phosphoproteomic studies [41,42]. These are thought to be mainly due to auto-phosphorylation of some parasite protein kinases within their activation loop [42]. Whether *Pf*Shelph2 might help regulate the enzymatic activity of some of these kinases remains to be explored. *Pf*Shelph2 could also dephosphorylate host cell proteins. Indeed, it has been well described that *P*. *falciparum* induces vast tyrosine and serine phosphorylation changes of RBCs proteins during its intracellular growth [43]. Moreover, mounting evidence supports the view where the interaction between free merozoite and erythrocyte triggers phosphorylation changes of RBC proteins [44–47] that may directly or indirectly modify the biophysical properties of the RBC membrane, as the phosphorylative status of RBC skeletal proteins affects erythrocyte deformability [48]. For instance, binding of *Pf*EBA175 to its RBC receptor glycophorin A was recently shown to induce significant modifications of the erythrocyte membrane deformability, essential for invasion [44,47]. Similarly, interaction between the adhesin *Pf*Rh5 and its host receptor Basigin triggers phosphorylations of numerous RBC cytoskeletal proteins including dematin, band 4.1, Ankyrin and spectrin [45]. Fernandez-Pol *et al*. already postulated that *Pf*Shelph2 may participate in the association/disassociation of Band3 from the underlying cytoskeleton during invasion based on its capacity to dephosphorylate Band 3 *in vitro* [18], but direct proof of *Pf*Shelph2 discharge in the RBC is still missing. The lack of detectable invasion defect in absence of *Pf*Shelph2 suggests that its putative function in dephosphorylating RBC substrates might not be of primary importance for invasion, although compensatory PPs of parasitic or erythrocytic origin might also substitute for *Pf*Shelph2.

Understanding the complex interplay between parasite kinases and PPs during *Plasmodium* life cycle still remains a challenge. To achieve this goal, we need to undertake a systematic functional characterization of its PPs but this task might be complicated by functional redundancy. In this context, the advent of CRISPR-Cas9 technology will be an invaluable tool to generate multi-knock-out or multi-edited parasite lines.

## Supporting information

S1 Table Primers used in this study. (PDF)

S1 Fig Verification of *Pf*Shelph2-HA_3_ edition by sequencing. (A) Sequencing showing *Pf*3D7 sequence (top) that corresponds to the guide RNA sequence followed by the PAM, and the related sequence in *Pf*Shelph2*-HA3 parasites (bottom) carrying the desired shield mutations without affecting the protein sequence. (B) Sequencing showing the 3’end of *shelph2* CDS in *Pf*3D7 (top), and the related sequence in *Pf*Shelph2-HA_3_ parasites (bottom) showing the successful in frame integration of the linker and HA_3_ tag.
(TIF)
