# Supplementary material for: Shelph2, a bacterial-like phosphatase of the malaria parasite Plasmodium falciparum, is dispensable during asexual blood stage
Source: PLoS One. 2017 Oct 26;12(10):e0187073. doi: 10.1371/journal.pone.0187073 (PMC5658161; doi:10.1371/journal.pone.0187073)
Supplement: S1 Table — (PDF) [file pone.0187073.s001.pdf]

S1 Table

Cloning primers		
**Primer name**	**Sequence (5’-3’) – Restriction site in bold**	**Restriction Site**
MLa33	TAAGTCCTCC**ACTAGT**GGAAGTGGAGGACGGGAATT	SpeI
MLa32	CGGAAGATA**GGCGCGCC**TTAGGCATAATCTGGAACATCG	AscI
MLa40	TATGCCTAA**GGCGCGCC**TACCTTTCATCATTTAAAAGGTCTC	AscI
MLa41	CAATGGCCCCTTT**CCGCGG**AGTAAAGCTTTACATATTCATTAAAAAG	SacII
MLa3	CGCCTCGAGATGAATATATCATATTTAAGGAATTTTTC	
MLa4	CGCGGTACCTATATCGGAATTTATATAATTTACTTTATATG	
MLa79	CTTCCTTATTATGCTAAAAGAGGTATTGATTATATAAATGATG	
MLa80	CATCATTTATATAATCAATACCTCTTTTAGCATAATAAGGAAG	
MLa59	CGCGGGGAGG**ACTAGT**CATTAGGGAAAATGTGTTCGTG	SpeI
MLa45	CTCCACTTCC**ACTAGT**TATATCGGAATTTATATAATTTACTTTATATG	SpeI
MLa60	TTACAAAATG**CTTAAG**AGTAAAGCTTTACATATTCATTAAAAAG	AflII
MLa63	TAAGTATATAATATTCTTCCTTATTATGCTAAGCGGTTTTAGAGCTAGAA	
MLa64	TTCTAGCTCTAAAACCGCTTAGCATAATAAGGAAGAATATTATATACTTA	
MLa54	TTTTACCGTT**CCATGG**GTTGAAAAATTATTATTATTTTATGGTG	NcoI
MLa53	ATTAAATCTA**GAATTC**TTAGAACACATTTTCCCTAATGG	EcoRI
MLa50	TTACAAAATG**CTTAAG**TACCTTTCATCATTTAAAAGGTCTC	AflII
MLa51	AGCCGAAGAT**ACTAGT**GGAAATTAGTATAATGCCCATGAAGTC	SpeI
MLa1	CGC**CTCGAG**ATGAATGTAGACAAAATACTTTGG	XhoI
MLa2	CGC**GGTACC**CAAATCTTTAATTTTATGACTTAGAC	KpnI
MLa11	CGCCTCGAGATGAAGAGTTTGGAGAATAACG	
MLa12	CGCGGTACCCATAAAATGACATTTCCTAAGAC	
MLa13	CGCCTCGAGATGTGGAATAAATTAAATGATGC	
MLa14	CGCGGTACCTAAAAAATTAAAACATTTAACATTAGG	
MLa65	CTTCCTTATTATGCTAAGCG	
MLa99	CCTTTTAAATGATGAAAGGTATTTGATATCC	
ML1476	CAGCGTAGTCCGGGACGTCGTAC	
MLa115	CAAGTTTATTATACATCCTATACATTTACTTTAAACC	
MLa116	ACGATGCAGTTTAGCGAACC	
MLa117	TCCAATACTTTCCAATGTTTCATGG	
hDHFR	CCAGGTGTTCTCTCTGATGTCC	
**qPCR primers**		
**Primer name**	**Sequence (5’-3’)**	**Gene**
MLa218	TGGCTAACCATAATTACCTTTTTGG	*shelph1*
MLa219	CTCTCTACGTCCCCATGGAT	*shelph1*
MLa224	AAGTGCCACCTCAAAGAGTG	*PPKL*
MLa225	GCTTCTGGTGGACTTCCTTT	*PPKL*
MLa226	TGTACCACCAGCCTTACCAG	*FBA*
MLa227	TTCCTTGCCATGTGTTCAAT	*FBA*

Shlp2_F	TGCTAAGCGTGGTATTGATT	*shelph2*
Shlp2_R	CTGCAGCACGAGAAAAGTAT	*shelph2*
