# Supplementary figures and images for: Shelph2, a bacterial-like phosphatase of the malaria parasite Plasmodium falciparum, is dispensable during asexual blood stage
Source: PLoS One. 2017 Oct 26;12(10):e0187073. doi: 10.1371/journal.pone.0187073 (PMC5658161; doi:10.1371/journal.pone.0187073)

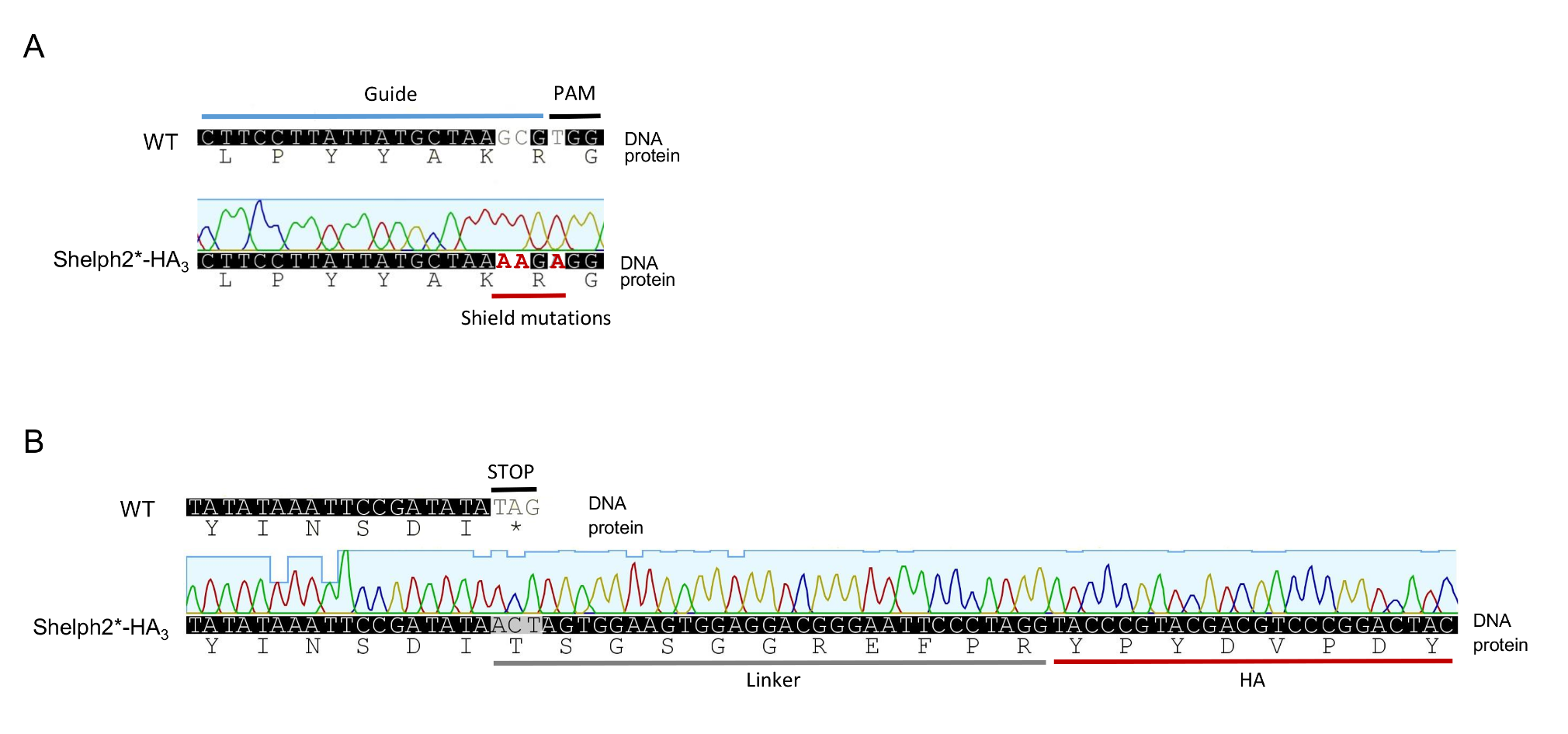

Supplement: S1 Fig — (A) Sequencing showing Pf3D7 sequence (top) that corresponds to the guide RNA sequence followed by the PAM, and the related sequence in PfShelph2*-HA3 parasites (bottom) carrying the desired shield mutations without affecting the protein sequence. (B) Sequencing showing the 3’end of shelph2 CDS in Pf3D7 (top), and the related sequence in PfShelph2-HA3 parasites (bottom) showing the successful in frame integration of the linker and HA3 tag. (TIF) [file pone.0187073.s002.tif]
